# Supplementary material for: RecA and Specialized Error-Prone DNA Polymerases Are Not Required for Mutagenesis and Antibiotic Resistance Induced by Fluoroquinolones in Pseudomonas aeruginosa
Source: Antibiotics (Basel). 2022 Feb 28;11(3):325. doi: 10.3390/antibiotics11030325 (PMC8944484; doi:10.3390/antibiotics11030325)
Supplement: Supplementary file 1 [file antibiotics-11-00325-s001.zip › antibiotics-1605351-supplementary.pdf]

RecA and specialized error-prone DNA polymerases are not required for mutagenesis and antibiotic resistance induced by fluoroquinolones in *Pseudomonas aeruginosa*

Jessica Mercolino, Alessandra Lo Sciuto, Maria Concetta Spinnato, Giordano Rampioni, and Francesco Imperi

SUPPLEMENTARY MATERIAL

Table S1. Bacterial strains and plasmids used in this study.

Strain or plasmid	Genotype and/or relevant characteristics	Reference or source
P. aeruginosa		
PAO1 (ATCC15692)	Prototroph	American type culture collection
Δ*recA*	PAO1 with an in-frame deletion of *recA* (PA3617)	[65]
Δ*recA recA*$^+$	Δ*recA* with a copy of *recA*, including its own promoter, inserted into the *attB* site of the chromosome	This work
Δ*polB*	PAO1 with an in-frame deletion of *polB* (PA1886)	[66]
Δ*dinB*	PAO1 with an in-frame deletion of *dinB* (PA0923)	[66]
Δ*imuC*	PAO1 with an in-frame deletion of *imuC* (PA0669)	[66]
Δ*polB* Δ*recA*	Δ*polB* deleted of *recA*	[66]
Δ*dinB* Δ*recA*	Δ*dinB* deleted of *recA*	[66]
Δ*imuC* Δ*recA*	Δ*imuC* deleted of *recA*	[66]
PAO1 CIP^R-1	Ciprofloxacin resistant spontaneous mutant of PAO1, selected on agar plate supplemented with 1 µg/mL ciprofloxacin (8×MIC)	This work
PAO1 CIP^R-1 Δ*recA*	PAO1 CIP^R-1 deleted of *recA*	This work
PAO1 CIP^R-2	Ciprofloxacin resistant spontaneous mutant of PAO1, selected on agar plate supplemented with 1 µg/mL ciprofloxacin (8×MIC)	This work
PAO1 CIP^R-2 Δ*recA*	PAO1 CIP^R-2 deleted of *recA*	This work
E. coli		
S17.1λ*pir*	*thi pro hsdRhsdM*$^+$*recA RP4-2-Tc::Mu-Km::Tn7 λpir,* Sm^R	[67]
**Plasmid**		
pBluescript II KS+	Cloning vector; ColE1 replicon; Ap^R	Stratagene
pDM4Δ*recA*	*sacB*-based suicide construct for the in-frame deletion of *recA* by homologous recombination	[65]
pDM4Δ*polA*	*sacB*-based suicide construct for the in-frame deletion of *polA* (PA5493) by homologous recombination	[66]
pDM4Δ*polB*	*sacB*-based suicide construct for the in-frame deletion of *polB* by homologous recombination	[66]
pDM4Δ*dinB*	*sacB*-based suicide construct for the in-frame deletion of *dinB* by homologous recombination	[66]
pDM4Δ*imuC*	*sacB*-based suicide construct for the in-frame deletion of *imuC* by homologous recombination	[66]
pDM4Δ*rmsA*	*sacB*-based suicide construct for the in-frame deletion of *rsmA* by homologous recombination	[38]

mini-CTX1	Self-proficient integration vector, Ω-*FRT*-*attP*-MCS, *ori*, *int*, *oriT*; Tc^R	[37]
mini-CTX1*recA*	mini-CTX1 derivative carrying the *P. aeruginosa recA* gene with its own promoter	This study
pFLP2	Broad-host-range plasmid expressing the Flp recombinase, *sacB*; Ap^R/Cb^R	[36]

Table S2. Primers used in this study.

**Primer name**	**Sequence (5'→3')**[a]	**Restriction site**	**Application**
*recA*_mini-CTX1_FW	cgcaagcTTCGGAACATTCTTCCCGTC	HindIII	Generation of
*recA*_mini-CTX1_RV	ccggatCCTCAATCGGCTTCGGCG	BamHI	mini-CTX1*recA*
*recN*_RT_FW	AGGAGCAGAAGACCCTGAGC		qRT-PCR
*recN*_RT_RV	GACAACAGGTTGACCGCTTC		
*recX*_RT_FW	CCGCTATCTCGAAAGCTTCA		
*recX*_RT_RV	CGCACTCCAGTCGACCTC		
*lexA*_RT_FW	GGCATGAGCATGAAGGACAT		
*lexA*_RT_RV	GAGCGAACTCAGGGTTTTCC		
*imuB*_RT_FW	GCCATGCTCTTCGAACTGAC		
*imuB*_RT_RV	TGTTCCCAGCCGAGATACTG		
*dinB*_RT_FW	GACTGTTTCTATGCCGCCCT		
*dinB*_RT_RV	CAGATCGGGACACAGCTTGA		
*polA*_RT_FW	TCGATTTCCTCGCCCTGATG		
*polA*_RT_RV	TCGCGATTTTCCTCGAGCTT		
*polB*_RT_FW	CTCGACTTCCTCCTCGAACG		
*polB*_RT_RV	CTTGTAGTCGAGCACCAGCA		
*polC*_RT_FW	GGTTATCGCAACCTCACCGA		
*polC*_RT_RV	GGAACACCTCCATCCACTCG		
*rpoD*_RT_FW	GGGCGAAGAAGGAAATGGTC		
*rpoD*_RT_RV	CAGGTGGCGTAGGTGGAGAA		
M13FW	GTTTTCCCAGTCACGAC		Sequencing of
M13RV	CAGGAAACAGCTATGAC		pBS constructs

[a] The restriction site used for cloning is underlined in the primer sequence.

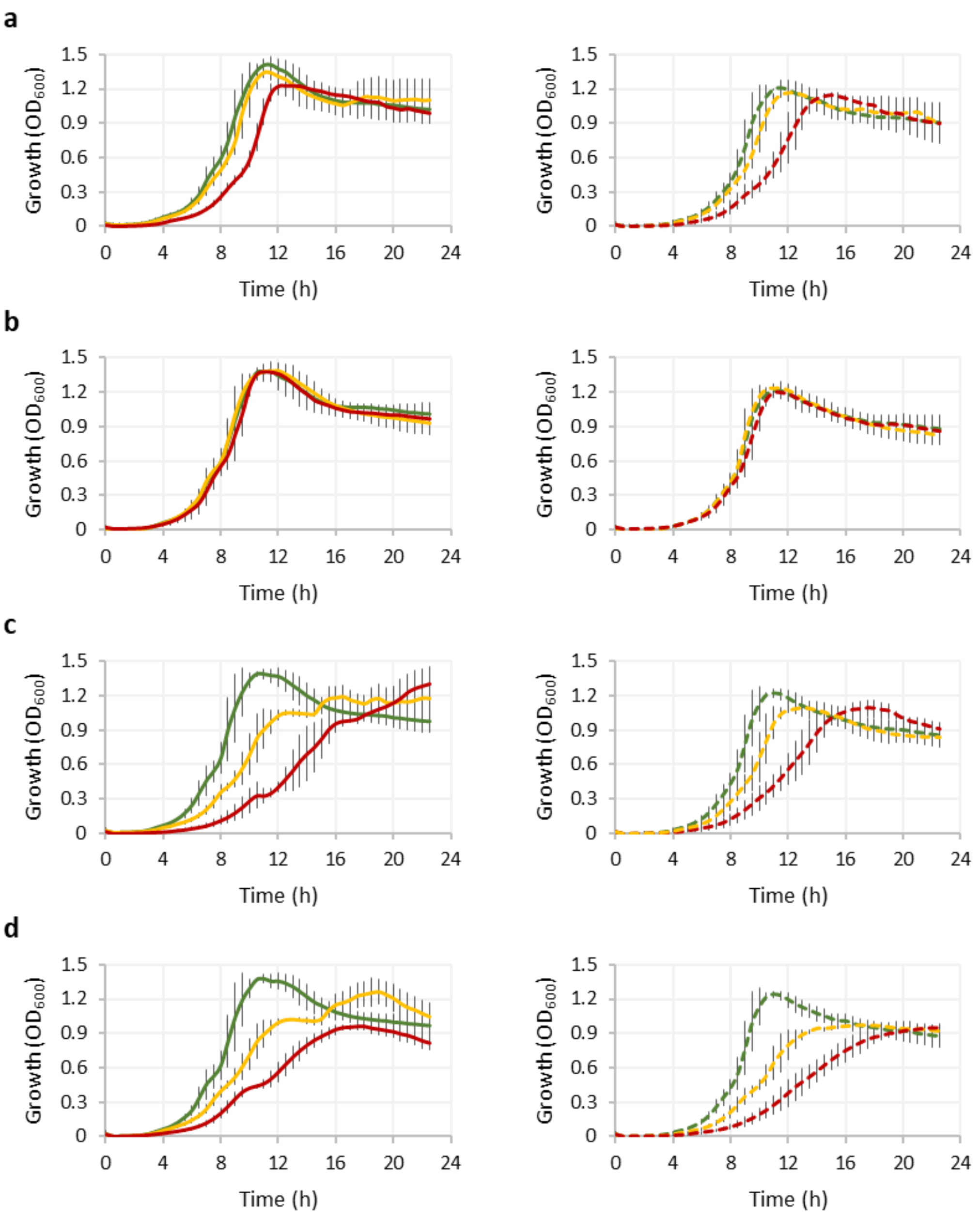

Figure S1. Growth curves of *P. aeruginosa* PAO1 (left panels, solid lines) and the Δ*recA* mutant (right panels, dotted lines) cultured in MH at 37°C in microtiter plates in the absence (green lines) or in the presence of (**a**) gentamicin, (**b**) colistin, (**c**) ofloxacin or (**d**) ciprofloxacin at 0.125×MIC (orange lines) or 0.25×MIC (red lines). Values are the mean (± SD) of three independent experiments.
